# Impact of myelofibrosis on patients with myelodysplastic syndromes following allogeneic hematopoietic stem cell transplantation

**DOI:** 10.1186/s12967-024-05080-3

**Published:** 2024-03-13

**Authors:** Panpan Zhu, Xiaoyu Lai, Lizhen Liu, Jimin Shi, Jian Yu, Yanmin Zhao, Luxin Yang, Tingting Yang, Weiyan Zheng, Jie Sun, Wenjun Wu, Yi Zhao, Zhen Cai, He Huang, Yi Luo

**Affiliations:** 1 Bone Marrow Transplantation Center of The First Affiliated Hospital & Liangzhu Laboratory, Zhejiang University School of Medicine, Hangzhou, 311121 China; 2 Institute of Hematology, Zhejiang University, Hangzhou, 311121 China; 3 Zhejiang Province Engineering Laboratory for Stem Cell and Immunity Therapy, Hangzhou, 311121 China

**Keywords:** Myelofibrosis, Hematopoietic stem cell transplantation, Myelodysplastic syndromes

## Abstract

**Background:**

The prognostic significance of myelofibrosis (MF) grade in patients with myelodysplastic syndrome (MDS) following an allogeneic hematopoietic stem cell transplantation (allo-HSCT) remains elusive.

**Methods:**

We retrospectively analyzed data from 153 patients with MDS who underwent allo-HSCT and divided the patients into the MF-0/1 (N = 119) and MF-2/3 (N = 34) cohorts to explore the impact of MF on outcomes of allo-HSCT.

**Results:**

The 2-year rates of relapse, non-relapse mortality (NRM), overall survival (OS), and progression-free survival (PFS) were 10.9% (95% confidence interval [CI] 5.9%–17.7%), 16.3% (95% CI 10.2%–23.6%), 76.6% (95% CI 69.0%–85.1%), and 72.8% (95% CI 65.0%–81.5%) in the MF-0/1 cohort, and 16.9% (95% CI 5.8%–32.9%), 14.7% (95% CI 5.3%–28.7%), 71.8% (95% CI 57.6%–89.6%), and 68.4% (95% CI 53.6%–87.2%) in the MF-2/3 cohort, respectively. No significant difference in the outcomes of allo-HSCT was observed between the two cohorts. Both univariate and multivariate analyses confirmed that MF-2/3 in patients with MDS had no effect on the prognosis of transplantation. In addition, major/bidirectional ABO blood type between donors and recipients was an independent risk factor for OS (hazard ratio [HR], 2.55; 95% CI 1.25–5.21; *P* = 0.010) and PFS (HR, 2.21; 95% CI 1.10–4.42; *P* = 0.025) in the multivariate analysis. In the subgroup of patients diagnosed with MDS with increased blasts (MDS-IB), it was consistently demonstrated that the clinical outcomes of the MF-2/3 cohort were comparable with those of the MF-0/1 cohort. The risk factors for OS and PFS in patients with MDS-IB were non-complete remission at transplantation and major/bidirectional ABO blood type.

**Conclusions:**

In conclusion, MF grade had no significant effect on prognosis of allo-HSCT in patients diagnosed with MDS. Major/bidirectional ABO blood type should be carefully considered in the context of more than one available donor.

**Supplementary Information:**

The online version contains supplementary material available at 10.1186/s12967-024-05080-3.

## Background

Myelodysplastic syndrome (MDS) is characterized by a heterogeneous hematological malignancy with a wide spectrum of presentation and implications. MDS with myelofibrosis (MF) accounts for 10%–20% of patients with de novo MDS [1, 2]. A series of prognostic scoring systems was established to guide treatment strategies for patients with MDS, such as the Revised International Prognostic Scoring System (IPSS-R) [3] and Molecular International Prognostic Scoring System (IPSS-M) [4]. Notwithstanding that MF is not widely included in scoring systems and is not always considered when making treatment decisions, it has been confirmed to be an independent risk factor for prognosis of MDS cases without transplantations [2, 5]. As reported previously, moderate to severe MF in primary MDS is significantly associated with multilineage dysplasia, transfusion dependence, and severe cytopenia [6–8]. A large study including 2,624 patients with MDS revealed that grade 3 MF contributed to a decreased survival rate, irrespective of IPSS-R [9]. Compared with patients without MF, a poor response to azacitidine was observed in patients with MDS concurrent MF [10]. Recently, the fifth edition of the World Health Organization (WHO) classification identified MDS with fibrosis (MDS-f) as a subentity [11].

Historically, emerging studies have been conducted to investigate the impact of MF on clinical outcomes of MDS cases under circumstance of allogeneic hematopoietic stem cell transplantation (allo-HSCT). However, the studies were quite inconclusive. It was recommended that the influence of MF should be factored in when allo-HSCT was proposed [12]. Previous studies confirmed that MF was closely associated with delayed engraftment and an inferior event-free survival following allo-HSCT [13, 14]. However, a recent study revealed that allo-HSCT may overcome the detrimental impact of moderate-to-severe MF on prognosis in patients with MDS. A significantly superior survival was observed in the allo-HSCT cohort compared to those without allo-HSCT (2-year overall survival [OS] rate, 68.4% versus 19.2%) [15]. At the meanwhile, a few of studies indicated that the MF degree had no impact on prognosis of MDS in patients receiving allo-HSCT [16, 17]. Taken together, the influence of MF on prognosis of MDS following allo-HSCT remains controversial. Therefore, we conducted a retrospective study to investigate the clinical characteristics, outcomes, and impact of MF on prognosis in patients with MDS following allo-HSCT.

## Methods

### Patients

This retrospective study included all patients with primary MDS with known bone marrow (BM) histology who underwent allo-HSCT from March 2016 to December 2022 at Bone Marrow Transplantation, the First Affiliated Hospital, Zhejiang University School of Medicine. The diagnostic criteria for MDS were based on the fifth WHO classification [11]. Patients who progressed to acute myeloid leukemia (AML) before transplantation were excluded. Details of follow-up data were obtained from medical records and telephone follow-up. This study was conducted in accordance with the Declaration of Helsinki and approved by the Ethics Review Committee of the First Affiliated Hospital, Zhejiang University School of Medicine (approval no. IIT 20231103A).

### Transplantation procedure

As previously described, conditioning regimens incorporated myeloablative conditioning (MAC) comprising busulfan and cyclophosphamide and reduced-intensity conditioning (RIC) comprising fludarabine and busulfan [18, 19]. Graft-versus-host disease (GVHD) prophylaxis comprised cyclosporin A, methotrexate, and low dose mycophenolate mofetil. For allo-HSCT recipients with haploidentical donors, rabbit antithymocyte globulin (ATG-G [Genzyme, Cambridge, MA, USA] or ATG-F [Fresenius, Bad Homburg, Germany]) was administered. For those using unrelated donors, ATG-G was applied. T-cell-replete grafts from granulocyte colony-stimulation factor-primed peripheral blood were applied to all patients.

### Definitions

OS was defined as the period from transplantation to the last follow-up or death from any cause. Progression-free survival (PFS) was calculated from the date of transplantation to progressive disease, relapse from disease remission, or death from any cause [20]. Relapse was defined as BM blasts of ≥ 5%, recurrence of blasts in blood, development of extramedullary disease, or development of worsening cytopenias [20]. Non-relapse mortality (NRM) was defined as death owing to any cause without relapse. Acute GVHD and chronic GVHD were identified according to previously established criteria by Harris et al*.* [21] and Jagasia et al*.*[22], respectively. Complete remission (CR) was defined as a reduction in BM blast percentage to < 5% and improvement in peripheral blood counts with a hemoglobin level of ≥ 10 g/dL, a platelet count of ≥ 100 × 10^9^/L, and an absolute neutrophil count of ≥ 1.0 × 10^9^/L independent of baseline values [20].Measurable residual disease (MRD) positivity was defined as ≥ 0.1% of leukemia-associated immunophenotype and different from normal aberrant immunophenotype in the bone marrow by multiparameter flow cytometry [23]. According to MRD status, patients who were confirmed with CR could be further divided into MRD negative group (CR-MRD negative) and MRD positive group (CR-MRD positive). The grading of MF into four categories (0, 1, 2, and 3) was based on the European MF network criteria [24]. Cases with MF-2/3 were considered to have moderate to severe MF. Patients were diagnosed with “MDS-f” when they met the diagnostic criteria of MDS with increased blasts (MDS-IB; blasts, 5%–19% BM or 2%–19% peripheral blood) and BM biopsy indicated MF > 0, in the absence of other prominent myeloproliferative features. Disease risk stratification was categorized according to the IPSS-R [3], and IPSS-M [4]. The disease risk for allo-HSCT was determined by the refined disease risk index (DRI) [25].

### Statistical analysis

Comparison of numerical variables between groups was performed using Student’s *t*-test or the Mann–Whitney U test. Comparison of the distribution of categorical variables in different groups was conducted using either Fisher’s exact test or the χ2 test. Curves were constructed for OS and PFS using the Kaplan–Meier method and compared using a log-rank test. The cumulative incidence of engraftment, GVHD, relapse, NRM was computed in a competing risk framework using the Fine and Gray method. Univariate and multivariate analyses were performed using the Cox proportional hazards regression model. Factors with *P* < 0.2 in the univariate analyses and those with clinical significance were included in the final multivariate model. Hazard ratios (HRs) with 95% confidence intervals (CIs) were calculated. Statistical analysis was conducted using SPSS statistical software version 27.0 (SPSS, Chicago, IL, USA) and R language statistical software (version 3.4.3, http:// www.r-project.org). *P* values were two-sided and considered significant if < 0.05.

## Results

### Patient and transplantation characteristics

A total of 153 patients diagnosed with primary MDS underwent allo-HSCT and were retrospectively evaluated for clinical outcomes, with a median follow-up of 23.1 (range, 0.1–91.3) months. The characteristics of the patients and transplantations are shown in Table 1. There were 89 males and 64 females, with a median age of 47 (range, 19–66) years. A total of 121 patients were diagnosed with MDS-IB, consisting of 57 with MDS-IB1/2 and 64 with MDS-f. The subgroup with MDS-IB comprised 34 patients with CR with negative MRD (CR-MRD^neg^), 32 with CR with positive MRD (CR-MRD^pos^), and 55 with non-complete remission (NCR) at the time of transplantation (Additional file 1: Table S1). According to genetic abnormalities, 13 patients were diagnosed with MDS-bi*TP53*. In summary, 34 (22.2%) of 153 patients were diagnosed with MF-2/3. No significant differences in patient age, patient sex, IPSS-R, IPSS-M, refined DRI, pre-HSCT chemotherapy, donor age, donor sex, donor type, ABO blood compatibility between donors and patients, conditioning regimen, ATG, graft mononuclear cells, and graft CD34^+^ cells were noted between the MF-0/1 and MF-2/3 cohorts. A total of 102 patients received haploidentical donor transplantation, 26 received matched sibling donor transplantation, and 25 underwent unrelated donor transplantation. The median donor age was 32 (range, 15–59) years.

**Table 1:** Characteristics of patients with myelodysplastic syndrome receiving transplantation

Variables	MF-0/1 N = 119 (77.8%)	MF-2/3 N = 34 (22.2%)	P
Patient age, years	48 (19–66)	46 (23–64)	0.637
Patient sex, male/female	67/52	22/12	0.381
Bone marrow blasts			
< 5%	26 (21.8%)	6 (17.6%)	0.595
≥ 5%	93 (78.2%)	28 (82.4%)	
MDS subentities			
MDS-LB/h	26	6	NA
MDS-IB1/2	57	0	
MDS-f	36	28	
MDS-bi*TP53*, Yes/No	9/110	4/30	0.438
Cytogenetics			
Good/very good	61 (51.3%)	19 (55.9%)	0.893
Intermediate	31 (26.1%)	8 (23.5%)	
Poor/very poor	27 (22.7%)	7 (20.6%)	
IPSS-R			
Low/Intermediate	34 (28.6%)	9 (26.5%)	0.810
High/Very high	85 (71.4%)	25 (73.5%)	
IPSS-M			
Low/Moderate low	15 (12.6%)	2 (5.9%)	0.485
High/Moderate high	57 (47.9%)	16 (47.1%)	
Very high	47 (39.5%)	16 (47.1%)	
Pre-HSCT Chemotherapy			
HMAs only	49 (41.2%)	14 (41.2%)	0.603
HMAs + VEN	13 (10.9%)	4 (11.8%)	
HMAs + chemotherapy	21 (17.6%)	3 (8.8%)	
Other	36 (30.3%)	13 (38.2%)	
Refined DRI			
Intermediate	58 (48.7%)	20 (58.8%)	0.300
High	61 (51.3%)	14 (41.2%)	
Donor age, years	32 (12 – 55)	30 (15 – 59)	0.799
Donor sex, male / female	78 / 41	23 / 11	0.820
Donor type			
HID	80 (67.2%)	22 (64.7%)	0.725
MSD	21 (17.6%)	5 (14.7%)	
URD	18 (15.1%)	7 (20.6%)	
ABO blood type			
Compatible	59 (49.6%)	21 (61.8%)	0.313
Major/bidirectional	33 (27.7%)	9 (26.5%)	
Minor	27 (22.7%)	4 (11.8%)	
Conditioning regimen			
RIC	35 (29.4%)	11 (32.4%)	0.742
MAC	84 (70.6%)	23 (67.6%)	
ATG			
ATG-G	75 (63.0%)	20 (58.8%)	0.872
ATG-F	33 (27.7%)	10 (29.4%)	
None	11 (9.2%)	4 (11.8%)	
MNCs (10^8^/kg)	11.4 (4.4–39.0)	11.8 (6.3–31.3)	0.649
CD34^+^ cells (10^6^/kg)	5.3 (1.2–14.3)	5.6 (2.1–22.5)	0.417

*HMA* hypomethylation agent; *IPSS-M* Molecular International Prognostic Scoring System; *IPSS-R* Revised International Prognostic Scoring System; *HID* haploidentical donor; *MSD* matched sibling donor; *URD* unrelated donor; *RIC* reduced intensity conditioning; *MAC* myeloablative conditioning; *MNC* mononuclear cell; *NA* not applicable

### Gene mutation spectrum

**Fig. 1:**
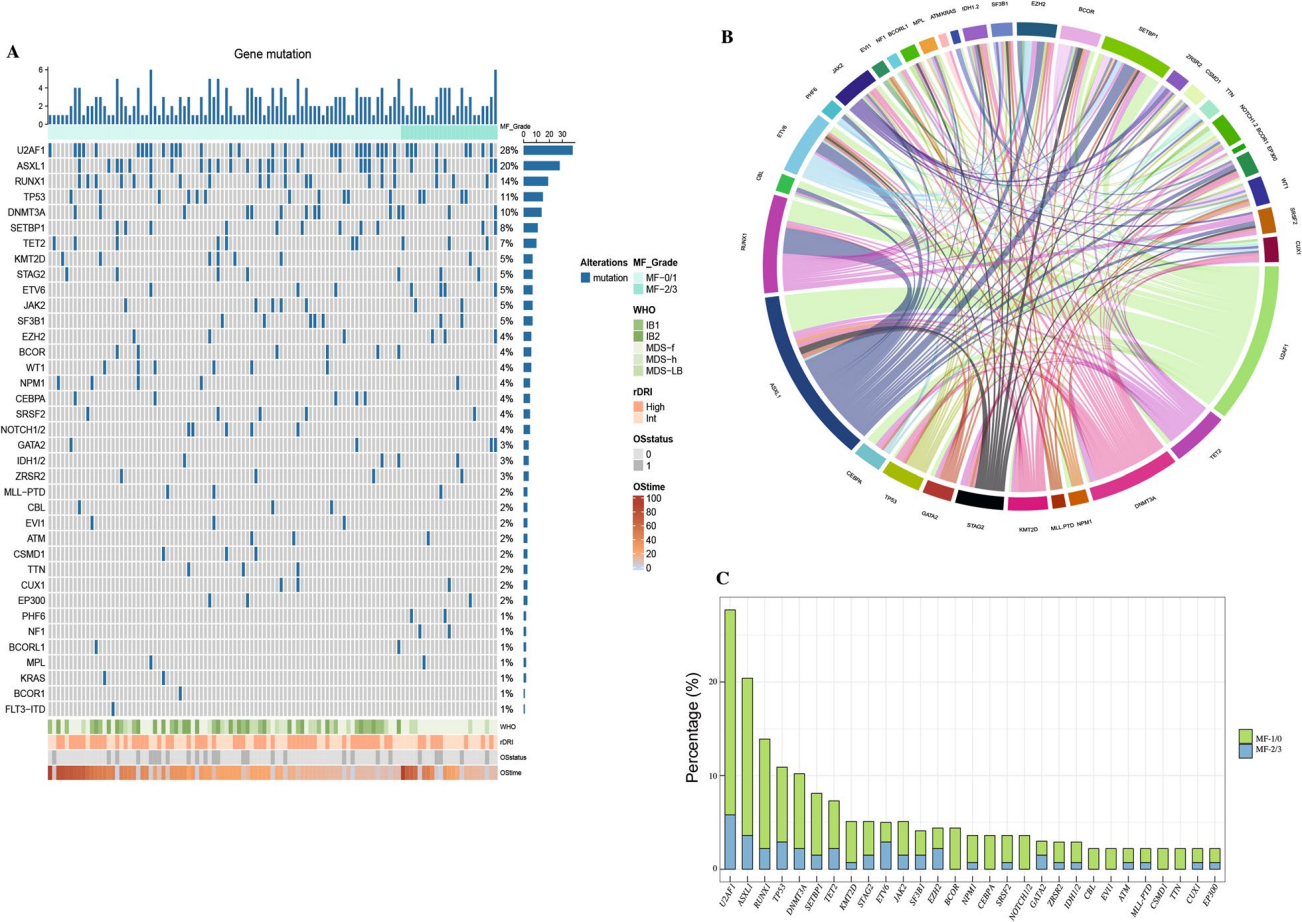
Mutation patterns in patients with myelodysplastic syndrome between the MF-0/1 and MF-2/3 cohorts, who were treated with allogeneic hematopoietic stem cell transplantation (N = 137). **A** Overview of gene mutation spectrum. The plot represents a graphical summary of the distribution of somatic lesions in sequenced genes across the set of patients. Columns represent samples and rows represent genes. Their number per sample and per gene is summarized on the top and on the left side of the plot, respectively. **B** Circos diagram depicts the relative frequency and pairwise co-occurrence of mutations. **C** Frequency of gene mutations according to the MF grade

Information on gene mutation was available in 137 patients. In total, 53 (38.7%) of 137 patients had zero to one oncogenic point mutations, 20 (14.6%) had two mutations, and 64 (46.7%) had more than three mutations. The gene mutation spectrum in patients with ≥ 3 gene alterations or those classified by IPSS-M is illustrated in Fig. 1. U2AF1 was the most frequently mutated gene (28%), followed by ASXL1 (20%), RUNX1 (14%), TP53 (11%), DNMT3A (10%), SETBP1 (8%), and TET2 (7%). No significant difference in the frequency of the seven gene alterations between the MF-0/1 and MF-2/3 cohorts was observed.

### Engraftment

The cumulative incidence rates of neutrophil engraftment at day 28 were 96.7% (95% CI 92.1%–98.7%), 97.5% (95% CI 92.0%–99.2%), and 94.1% (95% CI 70.9%– 98.9%) in the entire, MF-0/1, and MF-2/3 cohorts, respectively. The cumulative incidence rates of platelet engraftment at day 28 were 93.5% (95% CI 88.0%–96.5%), 93.3% (95%CI 86.8%–96.6%), and 94.1% (95%CI 71.0%–98.9%) in the entire, MF-0/1, and MF-2/3 cohorts, respectively. The median times to neutrophil and platelet engraftment were 12 (10–21) and 14 (8–35) days in the entire cohort, respectively. The median times to neutrophil engraftment in the MF-0/1 and MF-2/3 cohorts were 12 (10–21) and 14 (10–19) days (*P* = 0.966), respectively. The median times to platelet engraftment in the MF-0/1 and MF-2/3 cohorts were 13 (8–35) and 14 (10–26) days (*P* = 0.378), respectively.

### Graft-versus-host disease

**Fig. 2:**
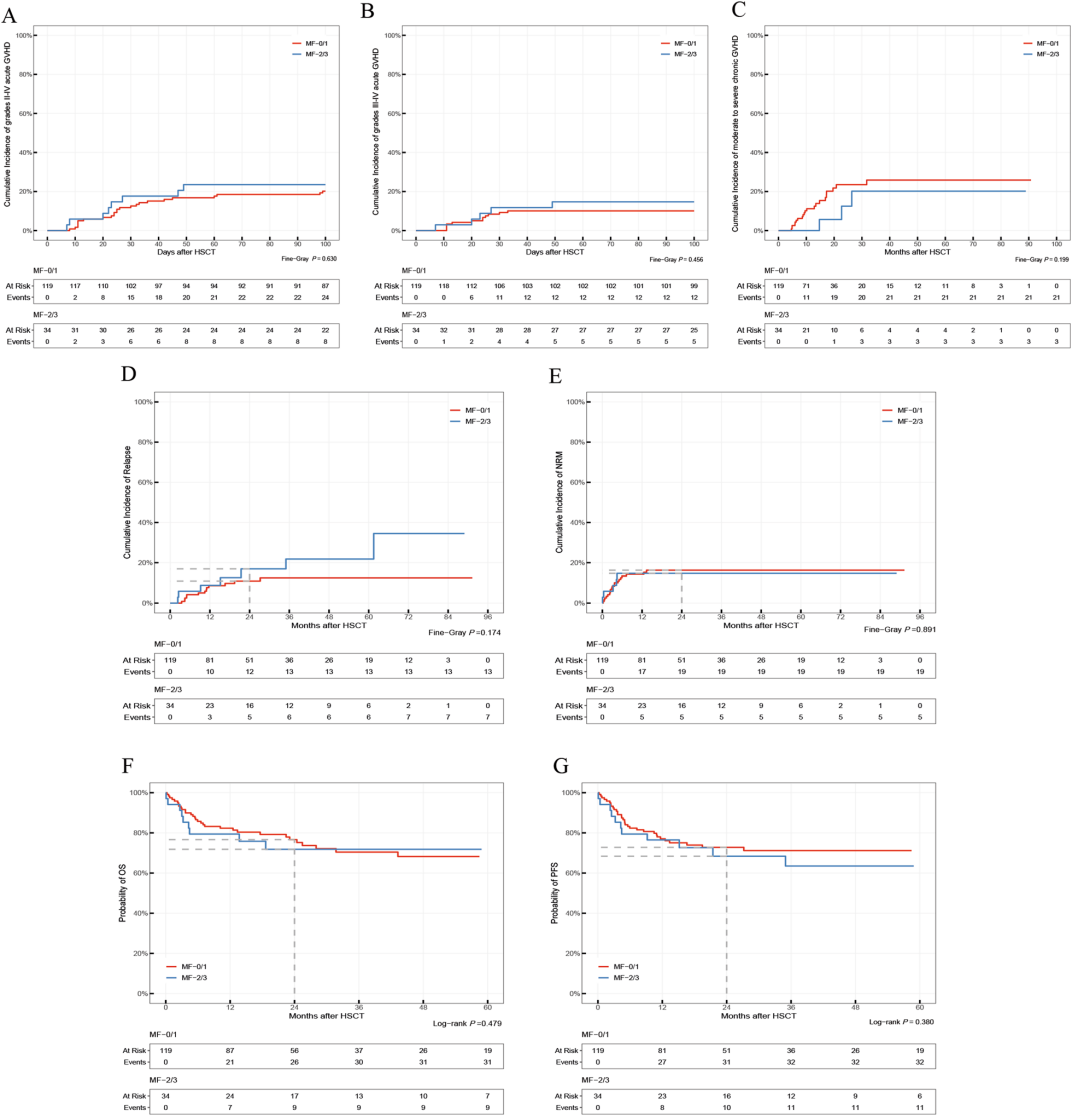
Clinical outcomes of the whole cases with MDS after transplantation in the MF-0/1 and MF-2/3 cohorts (N = 153). **A** Cumulative incidence rate of grade II–IV acute GVHD. **B** Cumulative incidence rate of grade III–IV acute GVHD. **C** Cumulative incidence rate of moderate-to-severe chronic GVHD. **D** Cumulative incidence rate of relapse. **E** Cumulative incidence rate of NRM. **F** OS probabilities. **G** PFS probabilities

The 100-day cumulative incidence rates of grade II–IV acute GVHD were 20.9% (95% CI 14.9–27.7%), 20.2% (95% CI 13.5–27.8%), and 23.5% (95% CI 10.9–38.9%) in the entire, MF-0/1, and MF-2/3 cohorts, respectively. The 100-day cumulative incidence rates of grade III–IV acute GVHD were 11.1% (95% CI 6.8–16.7%), 10.1% (95% CI 5.5–16.3%), and 14.7% (95% CI 5.3–28.7%) in the entire, MF-0/1, and MF-2/3 cohorts, respectively. The 2-year cumulative incidence rates of moderate to severe chronic GVHD were 21.2% (95% CI 13.5–30.0%), 23.5% (95% CI 14.7–33.5%), and 12.5% (95% CI 1.9–33.9%) in the entire, MF-0/1, and MF-2/3 cohorts, respectively. No significant difference in acute and chronic GVHD development was observed between the MF-0/1 and MF-2/3 cohorts (Fig. 2A–C).

### Relapse and NRM

**Fig. 3:**
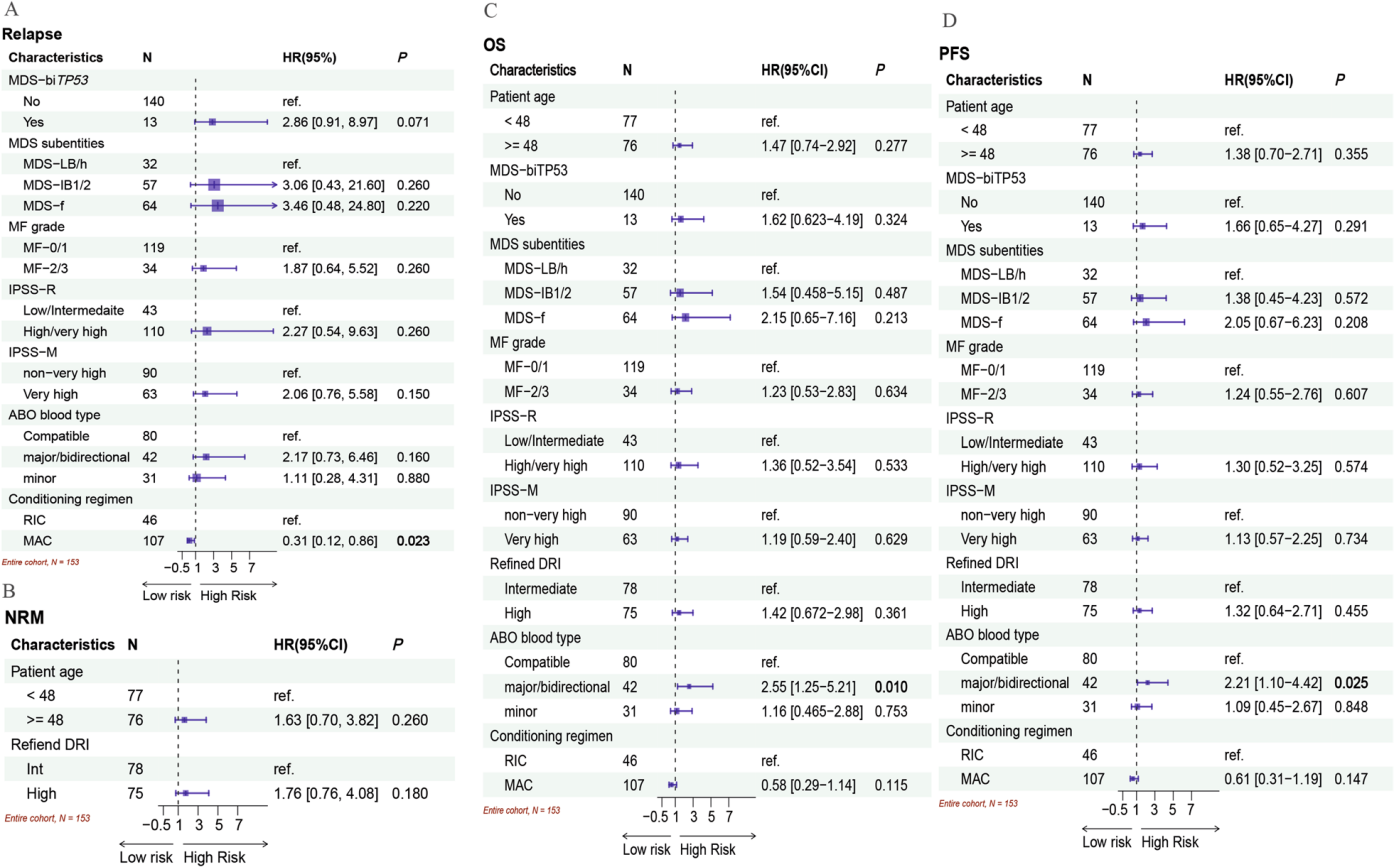
Multivariate analysis for clinical outcomes in the entire cohort (N = 153). **A** Relapse. **B** NRM. **C** OS. **D** PFS

The 2-year cumulative incidence rates of relapse in the entire, MF-0/1, and MF-2/3 cohorts were 10.2% (95% CI 5.8–16.1%), 10.0% (95% CI 5.3–16.6%), and 10.9% (95% CI 2.6–26.1%), respectively (Fig. 2D). The 2-year cumulative incidence rates of NRM were 16.6% (95% CI 11.1–23.0%), 16.3% (95% CI 10.2–23.6%), and 17.6% (95%CI 7.0–32.2%) in the entire, MF-0/1 and MF-2/3 cohorts, respectively (Fig. 2E). The univariate analysis indicated MDS-bi*TP53* and a very high risk of IPSS-M were significant risk factors for relapse (Additional file 1: Table S2). In the multivariate analysis, the MAC regimen was an independently favorable factor for disease relapse (HR, 0.31; 95% CI, 0.12–0.86, *P* = 0.023; Fig. 3A). As shown in the univariate and multivariate analyses (Additional file 1: Table S2, Fig. 2B), there were no critical variations for NRM. The impact of MF grade on relapse and NRM was determined by univariate and multivariate analyses (Additional file 1: Table S2, Fig. 3). The rates of relapse and NRM in the subgroups concerning the conditioning regimen are shown in Table 2.

**Table 2:** Survival rate at 2 years after allo-HSCT in the whole cohort (N = 153) according to the ABO blood type and conditioning regimens

Subgroups	MDS cohort (N = 153)			
	OS (95% CI)	PFS (95% CI)	Relapse (95% CI)	NRM (95% CI)
Donor-recipient ABO blood type				
Compatible	80.0% (71.4–89.8%)	76.3% (67.2–86.6%)	9.9% (4.3–18%)	14% (7.3–22%)
Major/bidirectional incompatible	64.0% (50.2–81.6%)	58.5% (44.4–77.0%)	19% (7.9–33%)	23% (11–37%)
Minor incompatible	79.2% (65.5–95.9%)	77.1% (63.6–93.6%)	10% (2.4–24%)	13% (4.0–27%)
*P*-value	0.088	**0.042**	0.276	0.481
Conditioning regimen				
RIC	68.6% (55.2–85.2%)	64.9% (51.7–81.5%)	17% (7.0–30%)	18% (8.4–31%)
MAC	78.3% (70.6–86.9%)	75.7% (66.6–83.7%)	10% (5.2–18%)	15% (9.0–22%)
*P*-value	0.091	0.113	**0.050**	0.774

Bold indicates the values with *P* < 0.05

**Fig. 4:**
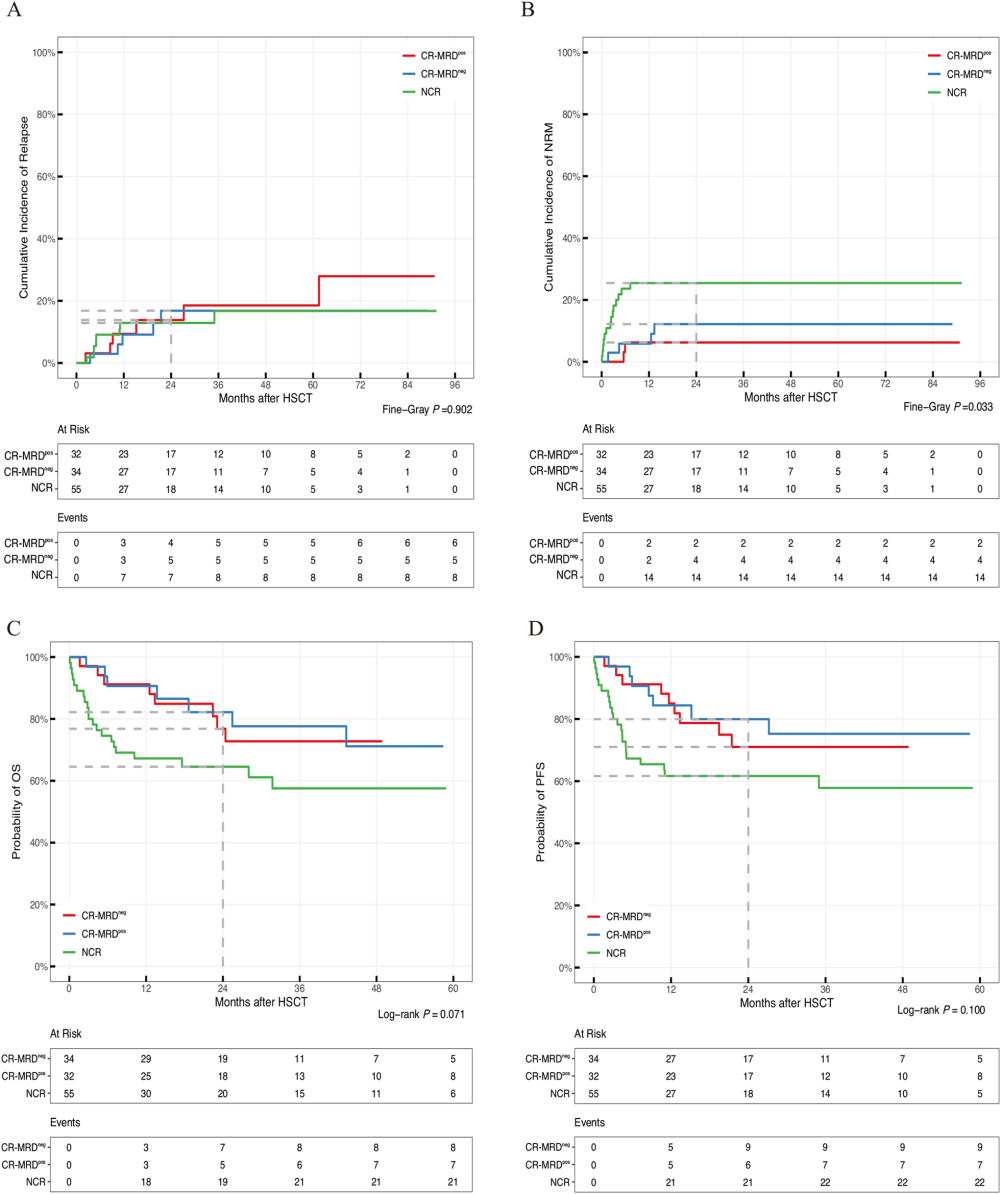
Clinical outcomes in patients with MDS-IB subgroup according to disease status at the time of transplantation (N = 121). **A** Cumulative incidence rate of relapse. **B** Cumulative incidence rate of NRM. **C** OS probabilities. **D** PFS probabilities

**Fig. 5:**
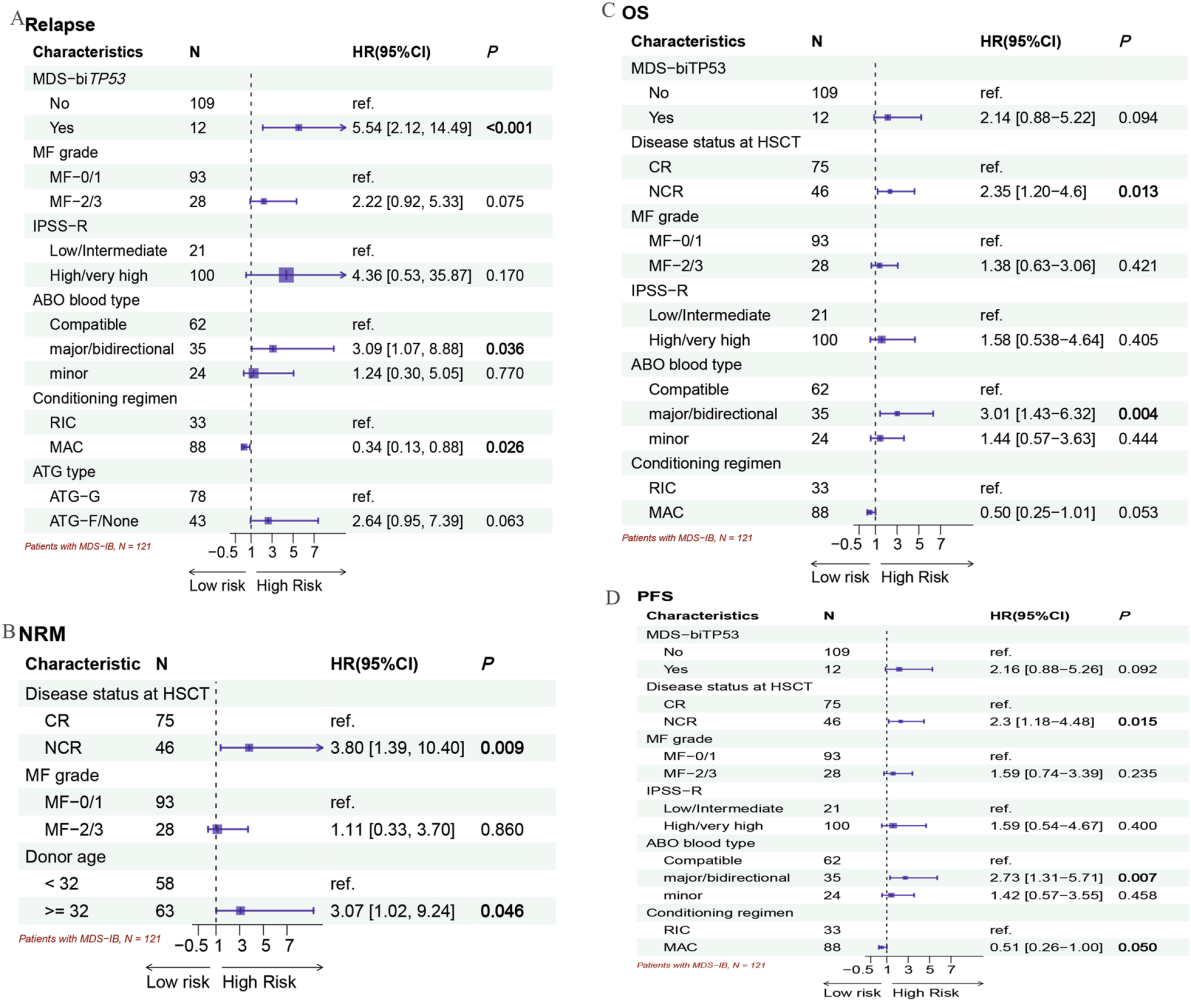
Multivariate analysis for clinical outcomes in patients with MDS-IB (N = 121). **A** Relapse. **B** NRM. **C** OS. **D** PFS

In the subgroup of patients with MSD-IB, the 2-year cumulative incidence rates of relapse between the MF-0/1 (13%; 95% CI 6.6–21%) and MF-2/3 (21%; 95%CI 7.0–40%) cohorts did not significantly differ (*P* = 0.133, Additional file 1: Fig. S1A). The NRM rate in the MF-0/1 cohort was 18% (95% CI 11–26%), which was similar to that in the MF-2/3 cohort (14%; 95% CI 4.4–30%; *P* = 0.754; Additional file 1: Fig. S1B). The 2-year cumulative incidence rates of relapse were 17% (95% CI 5.9–33%), 14% (95% CI 4.1–29%), and 13% (95% CI 5.6–23%) in the CR-MRD^neg^, CR-MRD^pos^, and NCR groups (*P* = 0.902, Fig. 4A), respectively. The 2-year cumulative incidence rates of NRM were 12% (95% CI 3.7–26%), 6.3% (95% CI 1.1–18%), and 25% (95% CI 15–38%) in the CR-MRD^neg^, CR-MRD^pos^, and NCR groups, respectively (*P* = 0.033, Fig. 4B). The univariate analysis for NRM and relapse was shown in Additional file 1: Table S3. The multivariate analysis for relapse (Fig. 5A) confirmed that the independent risk variations were MDS-bi*TP53* (HR, 5.54; 95% CI 2.12–14.49; *P* < 0.001) and major/directional incompatible ABO blood type between donors and recipients (HR, 3.09; 95% CI, 1.07–8.88; *P* = 0.036). A favorable factor for relapse was the MAC regimen (HR, 0.34; 95% CI 0.13–0.88; *P* = 0.026). Table 3 illustrated the incidences of relapse and NRM in cases with MDS-IB according to the ABO blood type and conditioning regimens. NCR at HSCT (HR, 3.80; 95% CI 1.49–10.40; *P* = 0.009) and older donor (aged ≥ 32 years) were risk factors for NRM (Fig. 5B). Similarly, MF had no effect on relapse or NRM in patients with MDS-IB receiving allo-HSCT (Fig. 5 and Additional file 1: Table S3).

**Table 3:** Survival rate at 2 years after allo-HSCT in patients diagnosed with MDS-IB (N = 121) according to the ABO blood type and conditioning regimens

Subgroups	MDS-IB cohort (N = 121)			
	OS (95% CI)	PFS (95% CI)	Relapse (95% CI)	NRM (95% CI)
Donor-recipient ABO blood type				
Compatible	79.1% (69.1–90.6%)	76.3% (66.1–88.1%)	11% (4.3–21%)	13% (6.0–23%)
Major/bidirectional incompatible	58.8% (43.3–79.9%)	51.7% (36.1–74.1%)	23% (9.5–40%)	25% (11–41%)
Minor incompatible	73.9% (57.8–94.5%)	70.6% (54.4–91.5%)	13% (3.0–30%)	17% (5.0–34%)
*P*-value	**0.039**	0.070	0.237	0.445
Conditioning regimen				
RIC	60.7% (43.8–84.0%)	55.0% (38.6–78.5%)	24% (9.7–43%)	21% (7.9–37%)
MAC	76.1% (67.4–85.9%)	73.1% (64.2–83.2%)	11% (5.3–19%)	16% (9.2–24%)
*P*-value	**0.032**	**0.034**	**0.006**	0.816

Bold indicates the values with *P* < 0.05

### Survival

The 2-year probabilities of OS in the entire, MF-0/1, and MF-2/3 cohorts were 76.4% (95% CI 69.7–83.8%), 76.6% (95% CI 69.0–85.1%), and 75.4% (95% CI 61.9–92.0%), respectively. The PFS rates at 2 years in the entire, MF-0/1, and MF-2/3 cohorts were 73.2% (95% CI 66.2–80.9%), 73.7% (95% CI 66.0–82.3%), and 71.4% (95% CI 57.0–89.5%), respectively. No significant differences in OS and PFS were observed between the MF-0/1 and MF-2/3 cohorts (Fig. 2F, G). The univariate analysis (Additional file 1: Table S2) revealed that the risk factors for OS included older patients age (≥ 48 years), MDS-f (*P* = 0.049) and major/bidirectional ABO blood type (*P* = 0.024). The multivariate analysis confirmed that major/bidirectional ABO blood type was an independent risk factor for OS (HR, 2.55; 95% CI 1.25–5.21; *P* = 0.010; Fig. 3) and PFS (HR, 2.21; 95% CI 1.10–4.42; *P* = 0.025; Fig. 3). The survival in subgroups concerning ABO blood type and conditioning regimen is presented in Table 2 (Additional file 1: Fig. S2A-B). MF in patients with MDS had no impact on the survival of allo-HSCT (Fig. 3 and Additional file 1: Table S2).

In the subgroup of patients with MSD-IB, the probability of 2-year OS was not significantly different, accounting for 73.4% (95% CI 64.5%–83.6%) in the MF-0/1 cohort and 69.0% (95% CI 53.0%–89.9%) in the MF-2/3 cohort (*P* = 0.516, Additional file 1: Fig. S1C). The 2-year PFS rate for patients with MDS-IB in the MF-0/1 cohort was 69.7% (95% CI 60.6%–80.0%), which was similar to that in the MF-2/3 cohort (64.9%; 95% CI 48.4%–87.1%, *P* = 0.394, Additional file 1: Fig. S1D). The 2-year probabilities of OS were 76.8% (95% CI 62.9%–93.8%), 82.2% (95% CI 68.9%–98.0%), and 64.5% (95% CI 52.8%–78.9%) in the CR-MRD^neg^, CR-MRD^pos^, and NCR groups, respectively (*P* = 0.071, Fig. 4C). The 2-year DFS rates in the CR-MRD^neg^, CR-MRD^pos^, and NCR groups were 71.0% (95% CI 56.5%–89.2%), 79.9% (95% CI 66.6%–96.0%), and 61.7% (95%CI 50.0%–76.0%), respectively (*P* = 0.100, Fig. 4D). Both univariate (Additional file 1: Table S3) and multivariate analyses (Fig. 5) revealed that NCR at HSCT and major/bidirectional ABO blood type were detrimental factors for OS and PFS in the MDS-IB subgroup. Compared with RIC, the MAC regimen was a favorable factor for PFS (HR, 0.51; 95% CI 0.26–1.00; *P* = 0.050). The clinical outcomes in subgroups concerning ABO blood type and conditioning regimen are illustrated in Table 3 (Additional file 1: Fig. S2C–D). Concordantly, no effect of MF on survival was identified in patients with MDS-IB receiving allo-HSCT (Fig. 5 and Additional file 1: Table S3).

## Discussion

In this retrospective study, we enrolled 153 patients diagnosed with primary MDS who underwent allo-HSCT to explore the effect of MF on the prognosis of transplantation. We found that patients with MF-2/3 had comparable survival with those with MF-0/1 under the circumstances of allo-HSCT (2-year OS, 71.8% vs. 76.6%, *P* = 0.479; 2-year PFS, 68.4% vs. 72.8%, *P* = 0.380). Major/bidirectional incompatible ABO blood type between donors and recipients resulted in inferior survival of patients with MDS than those with compatible or minor incompatible ABO blood type. Patients with MDS-IB may benefit from strategies for achieving CR at the time of transplantation or MAC regimen.

Historically, moderate to severe MF was considered an adverse factor for prognosis in patients diagnosed with MDS without allo-HSCT, which could be attributed to multilineage dysplasia, excess of blasts, and increased risk of early BM failure or leukemia transformation [2, 6, 26]. Similarly, some studies indicated that MF-2/3 was an adverse risk factor for outcomes in patients diagnosed with MDS who underwent allo-HSCT. As previously revealed, MF was an independent risk factor for OS rather than leukemia-free survival in the cohort after allo-HSCT [1].Wang et al*.* compared the survival in the MF-2/3 cohort and those in the MF-1 and MF-0 cohorts, and found an inferior estimated 3-year OS and PFS rate in patients with MF-2/3 following allo-HSCT (OS, 41.3% vs. 72.2% vs. 67.5%, *P* = 0.018; PFS, 44.8% vs. 72.8% vs. 68.8%, *P* = 0.018) [14]. They also indicated that MF predicted an inferior survival in individuals with ≥ 10% blasts in BM at diagnosis. In the subgroup with BM blasts of < 10%, cases of MF-2/3 had a comparable survival rate with those with MF-1 and MF-0 [14]. However, Scott et al*.* reported no significant differences in OS, relapse-free survival, and NRM between patients with MF and those without [16]. Remarkably, they found that MF was correlated with adverse transplantation prognosis in patients with advanced disease (int-2 or high-risk by IPSS) [16]. In our study, no association between MF grade and transplantation outcomes was observed, regardless of whether in the entire cohort or in the MDS-IB subgroup. The controversial results may be attributed to the available patients in our study, in which we excluded those transformed to AML before transplantation. Previous reports have revealed that MF is associated with a higher rate of leukemia transformation [14, 26], which may result in poor survival in patients with MDS. A previous study demonstrated that patients diagnosed with MDS concurrent with MF could achieve better survival in the allo-HSCT cohort than those without allo-HSCT [15]. In addition, they indicated no significant difference in survival between the MF-2/3 and MF-0/1 cohorts in allo-HSCT recipients. On the other hand, our study confirmed that MF has no significant impact on engraftment and NRM after transplantation. Nevertheless, some studies have found that moderate to severe MF results in delayed engraftment and transfusion independence, leading to a high risk of NRM following allo-HSCT [8, 14].

It remains controversial whether donor–recipient ABO blood type had a significant influence on transplantation outcomes. We demonstrated that major/bidirectional ABO incompatibility was a poor predictor of OS and PFS in patients diagnosed with MDS. In terms of the MDS-IB subgroup, a higher risk of relapse and NRM was observed in the major/bidirectional ABO-mismatched cohort. In line with our findings, Logan et al*.* confirmed that major ABO incompatibility predicted a higher risk of NRM and poor OS in patients with MDS and AML [27]. In the context of human leukocyte antigen-matched transplantation, a major ABO mismatch was independently associated with survival in patients with acute leukemia [28]. Moreover, major ABO incompatibility was correlated with delayed multilineage engraftment, leading to a relative long-term transfusion dependence and increased NRM [29]. Notwithstanding, Kimura et al*.* found that ABO blood type mismatch has lost its detrimental effect on clinical outcomes in unrelated BM transplantation [30]. In the myeloablative haploidentical transplantation, no association was noted between ABO blood type status and transplantation outcomes in patients diagnosed with hematological malignancies, where MDS accounted for 8% of the entire cohort [31]. Taken together, we hypothesized that the impact of ABO incompatibility could vary depending on the disease and donor type. For patients diagnosed with MDS, particularly for MDS-IB, the ABO blood type should be carefully considered when several candidate donors are available.

In our study, patients with MDS-IB benefited less when they were NCR at the time of transplantation or received the RIC regimen. NCR at allo-HSCT significantly increased the risk of NRM, leading to inferior OS and PFS. In alignment with our study, it was revealed that patients with MDS who achieved CR before allo-HSCT had improved outcomes [14]. Recently, it was confirmed that pre-HSCT MRD positivity was an independent risk factor for transplant outcomes in patients with MRD with excess blasts, along with the 3-year OS rates of 91.3% and 66.4% in the MRD-negative and MRD-positive cohorts, respectively [32]. Remarkably, reducing tumor burden was recommended in patients with ≥ 10% BM blasts, especially when RIC was planned [33]. The present study compared recipients with the RIC regimen, and a significantly lower incidence of relapse and superior survival was observed in those with the MAC regimen. Similarly, a long-term follow-up study by EBMT revealed that the 13-year relapse rate in patients with MDS was significantly higher in the RIC regimen than in the MAC regimen (48% vs. 31%; HR, 1.5; 95% CI 1.1–1.9; *P* = 0.04) [34]. In addition, a prospective phase III randomized study reported that patients with AML/MDS in the MAC cohort had superior OS compared with those in the RIC cohort [35]. However, a CIBMTR retrospective study revealed similar OS and PFS between the RIC and MAC regimens in patients with AML/MDS with a high to very high risk of refined DRI [36]. The presence of heterogeneous patients across different studies has posed challenges in explaining the contradictory results. To some extent, graft failure decreased in patients receiving the MAC regimen [37], which may facilitate superior survival in patients with MDS concurrent with MF.

Despite these important findings, our study is subject to several limitations. This study was conducted retrospectively so that enrolled patients and clinical data have an inherent bias. In addition, this study is featured by a relatively small size from a single center, which may have an influence on the results. Therefore, large prospective research conducted by multiple centers is required to assess the impact of clinical characteristics on transplantation outcomes in individuals diagnosed with MDS, particularly in cases concurrent with MF.

## Conclusions

In conclusion, our study confirmed that MF-2/3 had no impact on the prognosis of transplantation in patients diagnosed with MDS. Major/bidirectional ABO blood type should be carefully considered for allo-HSCT recipients with MDS in the context of more than one available donor. In the MDS-IB subgroup, reducing tumor burden to achieve CR at the time of transplantation or MAC regimen may improve post-HSCT OS and PFS. Prospective studies are needed to investigate the correlation between disease status, donor characteristics, transplantation features and transplant outcomes in patients diagnosed with MDS.

### Supplementary Information

**Additional file 1: Figure S1.** Clinical outcomes in patients with MDS-IB according to MF grade (N = 121). **A** Cumulative incidence rate of relapse. **B** Cumulative incidence rate of NRM. **C** OS probabilities. **D** PFS probabilities. **Figure S2. **Clinical outcomes after transplantation according to ABO blood type between patients and donors. **A** OS probabilities in the entire cohort. **B** PFS probabilities in the entire cohort. **C** OS probabilities in patients with MDS-IB. **D** PFS probabilities in patients with MDS-IB. **Table S1.** Characteristics of patients with myelodysplastic syndrome with increased blasts (MDS-IB, N = 121). **Table S2.** Univariate analysis of clinical outcomes and contributing factors in the entire cohort (N = 153). **Table S3.** Univariate analysis of clinical outcomes and contributing factors in patients with myelodysplastic syndrome with increased blasts (MDS-IB, N = 121).

## Data Availability

The dataset used and analyzed in this study are available from the corresponding author on reasonable request.

## Abbreviations

CR: Complete remission
DRI: Disease risk index
GVHD: Graft-versus-host disease
HR: Hazard ratio
IPSS-M: Molecular International Prognostic Scoring System
IPSS-R: Revised International Prognostic Scoring System
MAC: Myeloablative conditioning
MDS: Myelodysplastic syndrome
MDS-f: Myelodysplastic syndrome with fibrosis
MDS-IB: Myelodysplastic syndrome with increased blasts
MF: Myelofibrosis
MRD: Measurable residual disease
NRM: Non-relapse mortality
OS: Overall survival
PFS: Progression-free survival
RIC: Reduced-intensity conditioning
WHO: World Health Organization
