# Supplementary material for: Impact of myelofibrosis on patients with myelodysplastic syndromes following allogeneic hematopoietic stem cell transplantation
Source: J Transl Med. 2024 Mar 13;22:275. doi: 10.1186/s12967-024-05080-3 (PMC10938659; doi:10.1186/s12967-024-05080-3)
Supplement: Supplementary file 1 — Additional file 1: Figure S1. Clinical outcomes in patients with MDS-IB according to MF grade (N = 121). A Cumulative incidence rate of relapse. B Cumulative incidence rate of NRM. C OS probabilities. D PFS probabilities. Figure S2. Clinical outcomes after transplantation according to ABO blood type between patients and donors. A OS probabilities in the entire cohort. B PFS probabilities in the entire cohort. C OS probabilities in patients with MDS-IB. D PFS probabilities in patients with MDS-IB. Table S1. Characteristics of patients with myelodysplastic syndrome with increased blasts (MDS-IB, N = 121). Table S2. Univariate analysis of clinical outcomes and contributing factors in the entire cohort (N = 153). Table S3. Univariate analysis of clinical outcomes and contributing factors in patients with myelodysplastic syndrome with increased blasts (MDS-IB, N = 121). [file 12967_2024_5080_MOESM1_ESM.docx]

**Additional file**

**Impact of myelofibrosis on patients with myelodysplastic syndromes following allogeneic hematopoietic stem cell transplantation**

Panpan Zhu^1,2,3,4^, Xiaoyu Lai^1,2,3,4^, Lizhen Liu^1,2,3,4^, Jimin Shi^1,2,3,4^, Jian Yu^1,2,3,4^, Yanmin Zhao^1,2,3,4^, Luxin Yang^1,2,3,4^, Tingting Yang^1,2,3,4^, Weiyan Zheng^1,2,3,4^, Jie Sun^1,2,3,4^, Wenjun Wu^1,2,3,4^, He Huang^1,2,3,4*^, Yi Luo^1,2,3,4*^

^1^ Bone Marrow Transplantation Center, the First Affiliated Hospital, Zhejiang University School of Medicine, Hangzhou 311121, China

^2^ Liangzhu Laboratory, Zhejiang University Medical Center, 1369 West Wenyi Road, Hangzhou 311121, China

^3^ Institute of Hematology, Zhejiang University, Hangzhou 311121, China

^4^ Zhejiang Province Engineering Laboratory for Stem Cell and Immunity Therapy, Hangzhou 311121, China

*: Corresponding authors:

**Yi Luo, Ph.D.**

Bone Marrow Transplantation Center, First Affiliated Hospital, Zhejiang University School of Medicine; Liangzhu Laboratory, Zhejiang University Medical Center, 1369 West Wenyi Road, Hangzhou 311121, China

Phone:086-571-87233801

Email:luoyijr@zju.edu.cn

**He Huang, Ph.D.**

Bone Marrow Transplantation Center, the First Affiliated Hospital, Zhejiang University School of Medicine; Liangzhu Laboratory, Zhejiang University Medical Center, 1369 West Wenyi Road, Hangzhou 311121, China

Phone:086-571-87233801

Email:huanghe@zju.edu.cn

**This file includes:**

Additional file 1: Figures 2

Additional file 1: Tables 3

**Additional file 1: Figures**

**
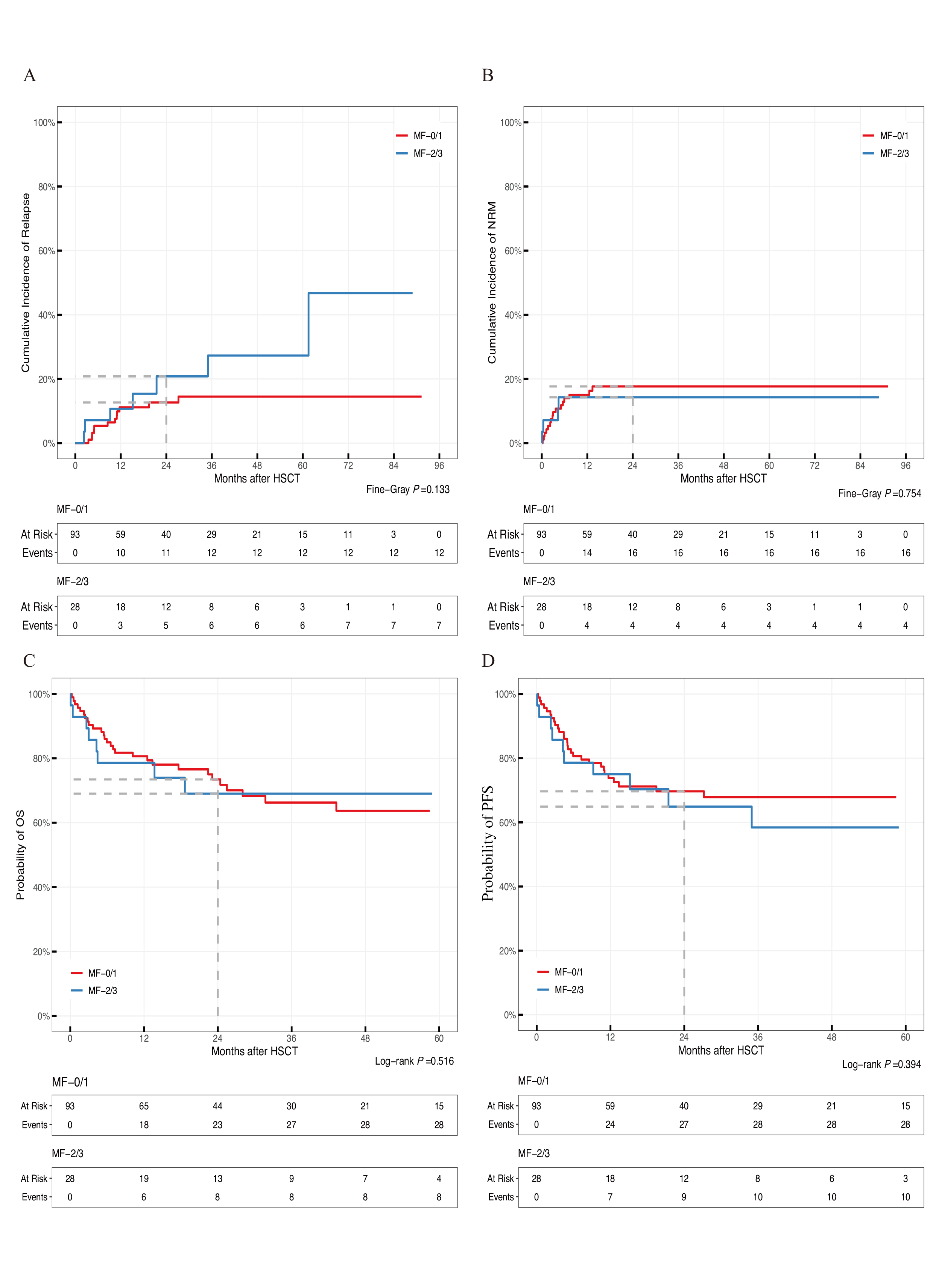
**

**Additional file 1: Figure. S1:** Clinical outcomes in patients with MDS-IB according to MF grade (N = 121). (**A**) Cumulative incidence rate of relapse. (**B**) Cumulative incidence rate of NRM. (**C**) OS probabilities. (**D**) PFS probabilities.

**
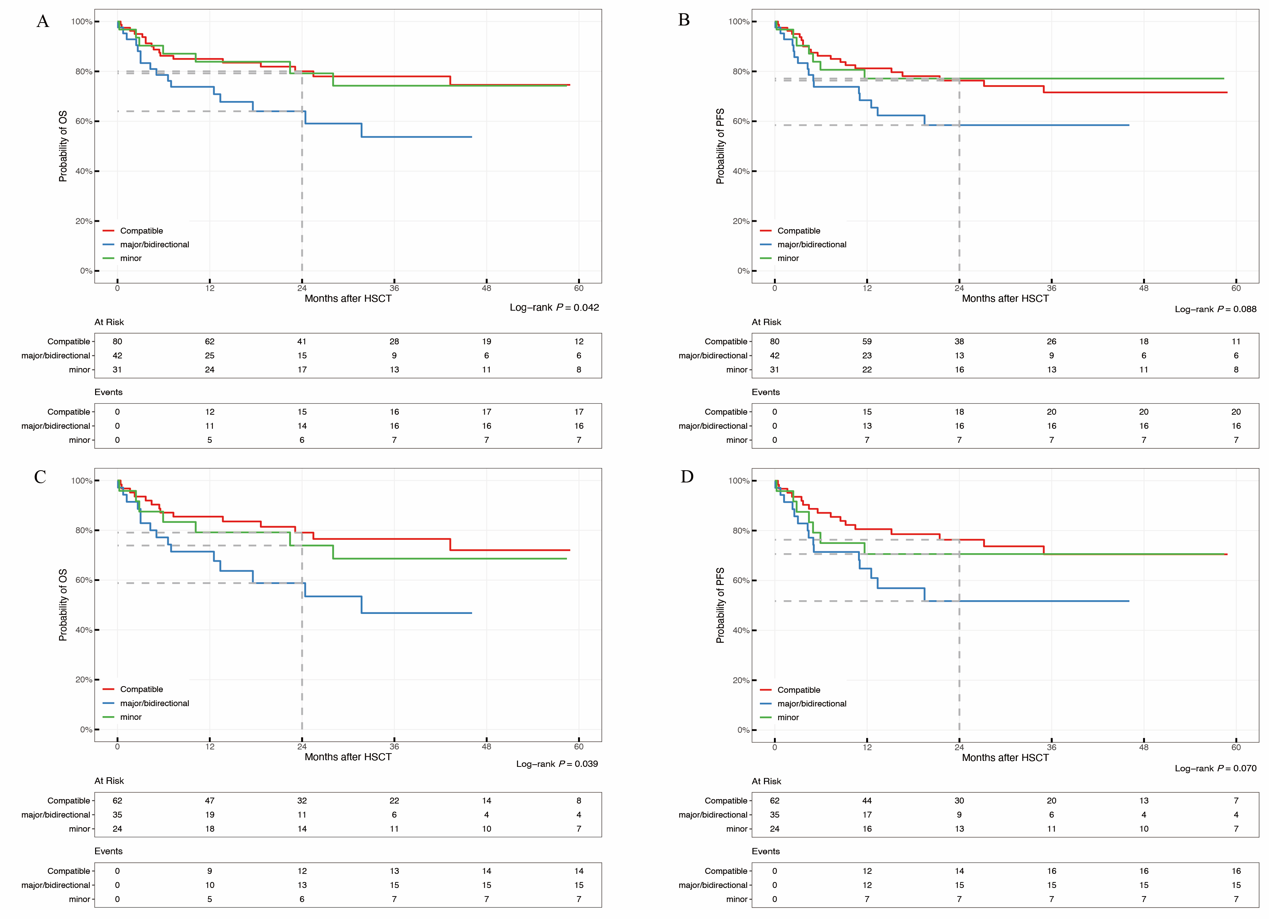
**

**Additional file 1: Figure. S2:** Clinical outcomes after transplantation according to ABO blood type between patients and donors. (**A**) OS probabilities in the entire cohort. (**B**) PFS probabilities in the entire cohort. (**C**) OS probabilities in patients with MDS-IB. (**D**) PFS probabilities in patients with MDS-IB.

**Additional file 1: Table S1. Characteristics of patients with myelodysplastic syndrome with increased blasts (MDS-IB, N = 121)**

| Variables | CR-MRD^neg^  N = 34 | CR-MRD^pos^  N = 32 | NCR  N = 55 | *P* |
| --- | --- | --- | --- | --- |
| Patient age, years | 48 (28 – 64) | 41 (21 – 66) | 50 (19 – 65) | 0.157 |
| Patient sex, male / female | 19 / 15 | 19 / 13 | 32 / 23 | 0.957 |
| Myelofibrosis |  |  |  | 0.245 |
| MF-0/1 | 25 (73.5%) | 22 (68.8%) | 46 (83.6%) |  |
| MF-2/3 | 9 (26.5%) | 10 (31.3%) | 9 (16.4%) |  |
| Cytogenetics |  |  |  | 0.484 |
| Good / very good | 16 (47.1%) | 18 (56.3%) | 28 (50.9%) |  |
| Intermediate | 10 (29.4%) | 6 (18.8%) | 19 (34.5%) |  |
| Poor / very poor | 8 (23.5%) | 8 (25.0%) | 8 (14.5%) |  |
| IPSS-R |  |  |  | 0.048 |
| Low / Intermediate | 5 (14.7%) | 10 (31.3%) | 6 (10.9%) |  |
| High / Very high | 29 (85.3%) | 22 (68.8%) | 49 (89.1%) |  |
| IPSS-M |  |  |  | 0.101 |
| Low / Moderate low | 1 (2.9%) | 6 (18.8%) | 3 (5.5%) |  |
| High / Moderate high | 18 (52.9%) | 12 (37.5%) | 22 (40.0%) |  |
| Very high | 15 (44.1%) | 14 (43.8%) | 30 (54.5%) |  |
| Chemotherapy protocol |  |  |  | 0.254 |
| HMAs only | 14 (41.2%) | 13 (40.6%) | 25 (45.5%) |  |
| HMAs + VEN | 8 (23.5%) | 3 (9.4%) | 5 (9.1%) |  |
| HMAs + chemotherapy | 8 (23.5%) | 6 (18.8%) | 9 (16.4%) |  |
| Other | 4 (11.8%) | 10 (31.3%) | 16 (29.1%) |  |
| Refined DRI |  |  |  | <0.001 |
| Intermediate | 26 (76.5%) | 24 (75.0%) | 51 (92.7%) |  |
| High | 8 (23.5%) | 8 (25.0%) | 4 (7.3%) |  |
| Donor age, years | 33 (13 – 50) | 35 (13 – 59) | 30 (15 – 55) | 0.234 |
| Donor sex, male / female | 20 / 14 | 26 / 6 | 36 / 19 | 0.132 |
| Donor type |  |  |  | 0.608 |
| HID | 22 (64.7%) | 20 (62.5%) | 42 (76.4%) |  |
| MSD | 5 (14.7%) | 6 (18.8%) | 5 (9.1%) |  |
| URD | 7 (20.6%) | 6 (18.8%) | 8 (14.5%) |  |
| ABO blood type |  |  |  | 0.240 |
| compatible | 20 (58.8%) | 15 (46.9%) | 27 (49.1%) |  |
| Major/bidirectional | 11 (32.4%) | 7 (21.9%) | 17 (30.9%) |  |
| Minor | 3 (8.8%) | 10 (31.3%) | 11 (20.0%) |  |
| Conditioning regimen |  |  |  | 0.989 |
| RIC | 9 (26.5%) | 9 (28.1%) | 15 (27.3%) |  |
| MAC | 25 (73.5%) | 23 (71.9%) | 40 (72.7%) |  |
| ATG |  |  |  | 0.758 |
| ATG-G | 21 (61.8%) | 19 (59.4%) | 38 (69.1%) |  |
| ATG-F | 9 (26.5%) | 9 (28.1%) | 14 (25.5%) |  |
| None | 4 (11.8%) | 4 (12.5%) | 3 (5.5%) |  |
| MNCs (10^8^/kg) | 15.0 (5.1 – 39.0) | 10.6 (4.4 – 30.1) | 11.0 (5.3 – 46.0) | 0.136 |
| CD34^+^ cells (10^6^/kg) | 5.02 (2.1 – 22.5) | 5.5 (1.2 – 14.3) | 5.4 (2.0 – 14.3) | 0.814 |

*MRD* measurable residual disease, *CR-MRD^neg^* complete remission with negative measurable residual disease, *MRD^pos^* complete remission with positive measurable residual disease, *HMA* hypomethylation agent, *VEN* venclexta, *IPSS-M* Molecular International Prognostic Scoring System, *IPSS-R* Revised International Prognostic Scoring System, *HID* haploidentical donor, *MSD* matched sibling donor, *URD* unrelated donor, *RIC* reduced-intensity conditioning, *MAC* myeloablative conditioning, *MF* myelofibrosis, *MNC* mononuclear cell

**Additional file 1: Table S2. Univariate analysis of clinical outcomes and contributing factors in the entire cohort (N = 153)**

| Variables | OS | | PFS | | Relapse | | NRM | |
| --- | --- | --- | --- | --- | --- | --- | --- | --- |
|  | *P* | HR (95% CI) | *P* | HR (95% CI) | *P* | HR (95% CI) | *P* | HR (95% CI) |
| Patient age (ref. < 48 years) | **0.050** | 1.87 (1.00 – 3.50) | **0.077** | 1.72 (0.94 – 3.16) | 0.410 | 1.43 (0.61 – 3.36) | **0.180** | 1.76 (0.77 – 4.01) |
| Patient sex (ref. female) |  |  |  |  |  |  |  |  |
| Male | 0.434 | 1.28 (0.69 – 2.39) | 0.366 | 1.33 (0.72 – 2.46) | 0.812 | 1.11 (0.45 – 2.72) | 0.364 | 1.48 (0.63 – 3.45) |
| MDS-bi*TP53* (ref. non- bi*TP53*) | **0.093** | 2.10 (0.88 – 4.99) | **0.109** | 2.03 (0.85 – 4.80) | **0.032** | 3.46 (1.11 – 10.8) | 0.940 | 0.95 (0.23 – 3.90) |
| MDS subentities (ref. MDS-LB/h) |  |  |  |  |  |  |  |  |
| MDS-IB-1/2 | **0.144** | 2.26 (0.76 – 6.77) | 0.203 | 1.92 (0.70 – 5.25) | **0.130** | 4.84 (0.62 – 37.5) | 0.820 | 1.15 (0.35 – 3.78) |
| MDS-f | **0.049** | 2.92 (1.00 – 8.47) | **0.054** | 2.59 (0.98 – 6.80) | **0.086** | 5.85 (0.78 – 44.1) | 0.420 | 1.59 (0.52 – 4.87) |
| MF (ref. MF-0/1) |  |  |  |  |  |  |  |  |
| MF-2/3 | 0.480 | 1.28 (0.64 – 2.55) | 0.382 | 1.34 (0.69 – 2.61) | **0.160** | 1.89 (0.78 – 4.63) | 0.890 | 0.93 (0.35 – 2.25) |
| IPSS-R (ref. Low / Intermediate) |  |  |  |  |  |  |  |  |
| High / Very high | **0.063** | 2.16 (0.96 – 4.86) | **0.085** | 1.96 (0.91 – 4.22) | **0.068** | 3.92 (0.91 – 17.0) | 0.640 | 1.24 (0.50 – 3.07) |
| IPSS-M (ref. non-very high) |  |  |  |  |  |  |  |  |
| Very high | **0.095** | 1.68 (0.91 – 3.07) | **0.135** | 1.57 (0.87 – 2.84) | **0.015** | 3.03 (1.24 – 7.40) | 0.730 | 0.86 (0.38 – 1.97) |
| Chemotherapy (ref. HMAs only) |  |  |  |  |  |  |  |  |
| HMAs + VEN | 0.717 | 0.82 (0.28 – 2.41) | 0.824 | 0.90 (0.34 – 2.38) | 0.650 | 0.71 (0.16 – 3.07) | 0.860 | 1.12 (0.31 – 4.06) |
| HMAs + chemotherapy | 0.745 | 0.87 (0.38 – 1.99) | 0.722 | 0.86 (0.38 – 1.95) | 0.660 | 0.79 (0.27 – 2.32) | 0.970 | 1.02 (0.33 – 3.20) |
| Other | 0.212 | 0.62 (0.29 – 1.32) | 0.140 | 0.57 (0.27 – 1.21) | 0.085 | 0.32 (0.09 – 1.17) | 0.800 | 0.88 (0.34 – 2.30) |
| Refined DRI (ref. Intermediate) |  |  |  |  |  |  |  |  |
| High | **0.082** | 1.32 (0.97 – 1.79) | **0.147** | 1.56 (0.86 – 2.83) | 0.850 | 1.09 (0.46 – 2.58) | **0.130** | 1.88 (0.84 – 4.25) |
| Donor age (ref. < 32 years) | 0.387 | 1.31 (0.71 – 2.42) | 0.401 | 1.29 (0.71 – 2.45) | 0.830 | 0.91 (0.38 – 2.16) | 0.210 | 1.68 (0.74 – 3.82) |
| Donor sex (ref. female) |  |  |  |  |  |  |  |  |
| Male | 0.673 | 1.15 (0.60 – 2.22) | 0.649 | 1.16 (0.61 – 2.19) | 0.650 | 1.25 (0.48 – 0.65) | 0.910 | 1.05 (0.45 – 2.44) |
| Donor type (ref. HID) |  |  |  |  |  |  |  |  |
| MSD / URD | 0.966 | 1.01 (0.53 – 1.94) | 0.738 | 0.90 (0.48 – 1.69) | 0.470 | 1.40 (0.57 – 3.44) | 0.310 | 0.63 (0.25 – 1.56) |
| ABO blood type (ref. compatible) |  |  |  |  |  |  |  |  |
| Major/bidirectional | **0.024** | 2.14 (1.10 – 4.17) | **0.054** | 1.89 (0.99 – 3.61) | **0.170** | 1.92 (0.76 – 4.84) | 0.270 | 1.63 (0.68 – 3.91) |
| Minor | 0.941 | 0.97 (0.40 – 2.32) | 0.795 | 0.89 (0.38 – 2.11) | 0.790 | 0.83 (0.22 – 3.13) | 0.920 | 0.95 (0.30 – 2.97) |
| Conditioning regimen (ref. RIC) |  |  |  |  |  |  |  |  |
| MAC | **0.097** | 0.59 (0.31 – 1.10) | **0.116** | 0.61 (0.33 – 1.13) | **0.066** | 0.43 (0.18 – 1.06) | 0.770 | 0.88 (0.39 – 2.03) |
| ATG (ref. ATG-G) |  |  |  |  |  |  |  |  |
| ATG-F / None | 0.760 | 0.91 (0.49 – 1.70) | 0.903 | 0.96 (0.52 – 1.77) | 0.320 | 1.57 (0.65 – 3.82) | 0.330 | 0.65 (0.27 –1.55) |

Bold indicates the values with *P* < 0.2

**Additional file 1: Table S3. Univariate analysis of clinical outcomes and contributing factors in patients with myelodysplastic syndrome with increased blasts (MDS-IB, N = 121)**

| Variables | OS | | PFS | | Relapse | | NRM | |
| --- | --- | --- | --- | --- | --- | --- | --- | --- |
|  | *P* | HR (95% CI) | *P* | HR (95% CI) | *P* | HR (95% CI) | *P* | HR (95% CI) |
| Patient age (ref. < 48 years) | 0.429 | 1.30 (0.68 – 2.49) | 0.476 | 1.26 (0.67 – 2.38) | 0.460 | 1.39 (0.59 – 3.34) | 0.890 | 1.06 (0.44 – 2.55) |
| Patient sex (ref. female) |  |  |  |  |  |  |  |  |
| Male | 0.495 | 1.26 (0.65 – 2.41) | 0.523 | 1.23 (0.65 – 2.35) | 0.940 | 1.03 (0.42 – 2.56) | 0.470 | 1.40 (0.56 – 3.50) |
| MDS-bi*TP53* (ref. non- bi*TP53*) | **0.131** | 1.96 (0.82 – 4.70) | **0.135** | 1.94 (0.81 – 4.64) | **0.059** | 3.02 (0.96 – 9.47) | 0.970 | 0.97 (0.23 – 4.03) |
| Disease status at HSCT (ref. CR) |  |  |  |  |  |  |  |  |
| NCR | **0.024** | 2.10 (1.10 – 4.00) | **0.036** | 1.97 (1.05 – 3.72) | 0.950 | 0.97 (0.39 – 2.43) | **0.017** | 2.89 (1.21 – 6.92) |
| MF (ref. MF-0/1) |  |  |  |  |  |  |  |  |
| MF-2/3 | 0.517 | 1.27 (0.62 – 2.62) | 0.396 | 1.35 (0.67 – 2.72) | **0.130** | 2.03 (0.82 – 5.02) | 0.760 | 0.84 (0.28 – 2.54) |
| IPSS-R (ref. Low / Intermediate) |  |  |  |  |  |  |  |  |
| High / Very high | **0.171** | 2.07 (0.73 – 5.83) | **0.173** | 2.05 (0.73 – 5.79) | **0.170** | 4.20 (0.53 – 33.36) | 0.700 | 1.26 (0.38 – 4.16) |
| Chemotherapy (ref. HMAs only) |  |  |  |  |  |  |  |  |
| HMAs + VEN | 0.684 | 0.80 (0.27 – 2.37) | 0.858 | 0.91 (0.34 – 2.47) | 0.600 | 0.67 (0.15 – 2.92) | 0.750 | 1.23 (0.33 – 4.58) |
| HMAs + chemotherapy | 0.635 | 0.82 (0.35 – 1.89) | 0.704 | 0.85 (0.37 – 1.96) | 0.570 | 0.73 (0.24 – 2.17) | 0.880 | 1.10 (0.34 – 3.56) |
| Other | 0.566 | 0.78 (0.34 – 1.80) | 0.516 | 0.76 (0.33 – 1.75) | 0.300 | 0.51 (0.14 – 1.86) | 0.880 | 1.09 (0.36 – 3.31) |
| Refined DRI (ref. Intermediate) |  |  |  |  |  |  |  |  |
| High | **0.070^#^** | 1.86 (0.95 – 3.64) | **0.114^#^** | 1.70 (0.88 – 3.27) | 0.850 | 0.92 (0.38 – 2.22) | **0.048^#^** | 2.71 (1.01 – 7.25) |
| Donor age (ref. < 32 years) | 0.292 | 1.42 (0.74 – 2.72) | 0.237 | 1.48 (0.77 – 2.82) | 0.850 | 0.92 (0.38 – 2.22) | **0.098** | 2.22 (0.86 – 5.74) |
| Donor sex (ref. female) |  |  |  |  |  |  |  |  |
| Male | 0.343 | 1.42 (0.69 – 2.93) | 0.414 | 0.86 (0.61 – 1.23) | 0.890 | 1.07 (0.41 – 2.79) | 0.430 | 1.50 (0.55 – 4.08) |
| Donor type (ref. HID) |  |  |  |  |  |  |  |  |
| MSD / URD | 0.555 | 1.23 (0.62 – 2.42) | 0.751 | 1.11 (0.57 – 2.17) | 0.220 | 1.78 (0.71 – 4.44) | 0.490 | 0.71 (0.26 – 1.90) |
| ABO blood type (ref. compatible) |  |  |  |  |  |  |  |  |
| Major/bidirectional | **0.018** | 2.34 (1.15 – 4.75) | **0.031** | 2.15 (1.07 – 4.32) | **0.140** | 2.05 (0.80 – 5.28) | 0.210 | 1.87 (0.71 – 4.95) |
| Minor | 0.796 | 1.13 (0.46 – 2.77) | 0.798 | 1.12 (0.46 – 2.74) | 0.870 | 0.89 (0.23 – 3.47) | 0.650 | 1.32 (0.40 – 4.39) |
| Conditioning regimen (ref. RIC) |  |  |  |  |  |  |  |  |
| MAC | **0.036** | 0.49 (0.25 – 0.95) | **0.038** | 0.50 (0.26 – 0.96) | **0.010** | 0.31 (0.13 – 0.76) | 0.820 | 0.90 (0.35 – 2.29) |
| ATG (ref. ATG-G) |  |  |  |  |  |  |  |  |
| ATG-F / None | 0.838 | 0.93 (0.48 – 1.81) | 0.879 | 1.05 (0.55 – 2.01) | **0.150** | 1.97 (0.79 – 4.92) | 0.280 | 0.58 (0.21 – 1.57) |

Bold indicates the values with *P* < 0.2

^#^ Refined DRI was based on disease status at transplantation and excluded in multivariate analysis.
